# Electrochemical modeling and evaluation for textile electrodes to skin

**DOI:** 10.1186/s12938-020-00772-5

**Published:** 2020-05-11

**Authors:** Jinzhong Song, Yu Zhang, Yijing Yang, Hao Liu, Tianshu Zhou, Kui Zhang, Fan Li, Zhi Xu, Qingjun Liu, Jingsong Li

**Affiliations:** 1 Key Laboratory for Biomedical Engineering of Ministry of Education, Engineering Research Center of EMR and Intelligent Expert System, Ministry of Education, College of Biomedical Engineering and Instrument Science, Zhejiang University, Hangzhou, 310027 China; 2 State Key Laboratory of Space Medicine Fundamentals and Application, China Astronaut Research and Training Center, Beijing, 100094 China; 3 School of Textiles, Tianjin Polytechnic University, Tianjin, 300387 China; 4 Research Center for Healthcare Data Science, Zhejiang Lab, Hangzhou, 311100 China

**Keywords:** Textile electrode, Skin–electrode electrochemical interface (SEEI), Skin–electrode electrochemical characteristic (SEEC), Simulated skin model (SSM), Electrochemical evaluation platform (EEP)

## Abstract

**Background:**

With the development of wearable health-monitoring technologies, a variety of textile electrodes have been produced and applied by researchers. However, there are no universal and effective methods even testing platforms for evaluating the skin–electrode electrochemical interface for textile electrodes because different human bodies have different skin characteristics.

**Methods:**

An electrochemical modeling and evaluation for textile electrodes to skin was proposed, and two electrochemical evaluation platforms (EEP) were set up based on two simulated skin models (SSM). First, skin–electrode electrochemical interface (SEEI) models for traditional wet electrodes and textile electrodes were analyzed. Based on the SEEI models and YY/T 0196-2005 (Chinese YY/T pharmaceutical industry standard for disposable ECG electrode), three skin–electrode electrochemical characteristics (SEEC), including skin–electrode static impedance (SESI), skin–electrode alternating current impedance (SEAI), and skin–electrode polarization voltage (SEPV), were proposed. Then, three electrochemical evaluation methods for textile electrodes to skin were proposed and analyzed, which were the correlation between SEEC and skin–electrode contact pressure (SECP), skin–electrode relative movement (SERM), and conduction loss of active signals (CLAS). Finally, an electrochemical evaluation platform was set up based on an active simulated skin model (ASSM) and passive simulated skin model (PSSM).

**Results:**

9 feature parameters based on the passive electrochemical evaluation platform (PEEP) and 11 feature parameters based on the active electrochemical evaluation platform (AEEP) were obtained for evaluating textile electrodes. And four kinds of textile electrode characteristics including SEEC, SECP, SERM, and CLAS were quantitatively measured based on the electrochemical evaluation platform, and the testing accuracy and range for these characteristics were measured separately. Finally, correlation between SEEC and SECP for 10 kinds of textile electrode samples was studied, and 14 electrochemical characteristics and four skin–electrode contact pressure characteristics were extracted. Experimental results showed that significant correlations were found between six SEEC characteristics and SECP characteristics, and the correlation coefficient between ACI_3 and USECP was the highest. And the polarization voltages of most dry electrode samples showed a downward trend with the increase of contact pressure.

**Conclusions:**

The electrochemical evaluation platform yielded effective experimental data and could provide strong support for the evaluation and application of textile electrodes, which was also effective in evaluating other bioelectric electrodes such as 3M electrode, stainless steel electrode, dry electrode and microneedle electrode.

## Background

Biomedical signals, such as electrocardiograms (ECGs), electroencephalograms (EEGs), electro-oculograms (EOGs), and electromyograms (EMGs), are widely used in patient monitoring, health examinations and family care, which provide an important basis for human health examinations [1, 2]. Biomedical signals are usually collected by wet electrodes, which are often used with a conductive paste to ensure good contact between the skin and electrodes. The approach has been widely recognized for its stability and fidelity in biomedical signal measurements. With the continuous improvement in people’s living standards, wearable health monitoring will become useful not only in the hospital but also at home, and a low medical supervisor load has become an important target in the development of biomedical signal acquisition technology. Signal extraction by traditional wet paste electrodes presents many problems, such as inconvenience and allergies [3].

Recently, a variety of wearable dry electrodes have attracted attention because they are used to obtain biomedical signals from human bodies with less skin pretreatment and conductive paste. Several types of dry electrodes have been produced, such as textile electrodes, noncontact electrodes, and microneedle electrodes. Textile electrodes as one kind of wearable dry biosensors were a form of dry electrodes with a textile structure and could be used to extract bioelectrical signals from the surface of human bodies, and it was easily integrated into wearable clothes used for health monitoring. Textile electrodes are comfortable, wearable, washable, and stable, which are divided into active and passive electrodes according to whether they can be integrated with signal recording and processing circuit units.

For active textile electrodes, impedance conversion circuits were usually applied to making input impedance smaller than passive electrodes [4]. Two kinds of textile electrodes for ECG monitoring were designed by Garey et al.: one design used polyurethane thin-film technology to enable direct contact between the conductive fabric part and the PCB, and the other involved inserting the movable electrode directly into a simple fabric circuit, which enabled miniaturization and low interference [5]. Another type of electrode was made on a planar-fashionable circuit board (P-FCB), and other components for measuring ECGs were soldered to the P-FCB, whose characteristics among printed metal plate electrodes, textile electrodes and microneedle electrodes had been systematically analyzed [6].

Textile electrodes with various structures have been developed, including woven, knitted, nonwoven, and embroidered structures. For textile electrodes, domestic and foreign researchers have used many kinds of metal materials, such as pure wires, metallized polymer filaments, and conductive textiles [7, 8]. Different textile processes with different conductive textile materials were used to develop textile electrodes: An electroless plating method was used to coat the surface of nylon filaments with gold, forming a conductive fiber thread [9]; pure unembroidered steel wire was used as an electrode material in a textile structure [10]; one textile electrode was made of silver-plated nylon fiber [11]; a copper-sputtering method was used to apply conductive treatment to the surface of a fabric [12, 13]; the surface of a nylon textile was subjected to silver treatment by vacuum sputtering [14, 15]; conductive fibers and conventional fibers were blended to make conductive yarns [16–18]. In addition, one kind of flexible dry electrode was used for long-term ECG measurements; PDMS (poly-dimethyl siloxane) films containing copper metal layers and a 3-mm-thick PDMS structure (including protruding parts) were studied by Baek et al.; and four conductive fibers on clothing were used for ECG detection [19, 20].

Textile electrodes commonly used in the market are usually Ag-plated textiles, which can easily cause a large impedance and a large polarization voltage in the electrode–skin interface [3, 21]. Textile electrodes often present many problems when used to collect biomedical signals, such as noise interference, a balance between human comfort and skin–electrode contact pressure, accurate measurement of electrochemical characters, including static impedance (SI), alternating current impedance (ACI), and polarization voltage (PV). The extraction of biomedical signals from the human body by textile electrodes needs less skin pretreatment and conductive paste than by traditional paste electrodes, and how to evaluate textile electrodes has become the focus of people’s attention. There were usually two electrochemical evaluation methods for textile electrodes, including direct detection method and reference detection method [3].

The direct detection method was thought that the impedance of the skin tissue is small (about 150 Ω), and two electrodes on the surface of skins were applied [9]. And this method was used to measure the skin–electrode contact impedance between dry electrodes and the skin behind the ear by Vojkan Mihjlovic, and it was found that the skin–electrode contact impedance was greatly affected by the contact pressure [10]. This method was also used to evaluate textile electrodes at the finger end by Antonio Lanata [11] and measure skin–electrode contact impedance by Dilpreet Buxi separately [12]. In addition, the reference detection method was used as a vector admittance measurement method for skin–electrode contact impedance evaluation with two reference electrodes, which was also used to analyze textile electrodes with four reference electrodes [13, 14].

However, the above methods could not evaluate quantitatively the electrochemical characteristics of the skin–electrode interface, and there are no universal and effective methods even testing devices for evaluating textile electrodes because different human bodies have different skin characteristics. Hence, an electrochemical skin modeling and evaluation for textile electrodes to skin was proposed in this research, and an evaluation platform based on simulated-skin models was developed.

## Results

### Textile electrode samples

**Fig. 1:**
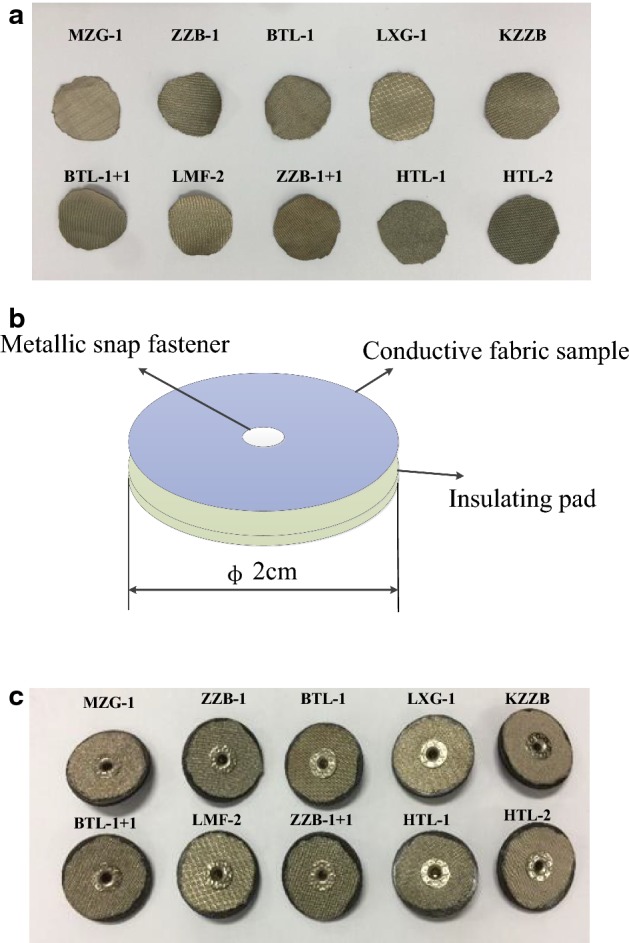
Textile samples. **a** Conductive fabric samples, **b** decomposition diagram for textile electrodes, **c** textile electrode samples

As shown in Fig. 1a, 10 kinds of conductive fabric samples with different plating processes and different textile processes were purchased from Qingdao Hengtong X-Silver Speciality Textile Co., Ltd, China, and they were cut into circles with a diameter of 2 cm. As shown in Fig. 1c, based on metallic snap fastener, insulating pad, and the fabric samples, 10 types of textile electrodes were fabricated, whose decomposition diagram is shown in Fig. 1b. Ten textile electrode samples are listed in Table 1, and textile electrode composition and technology are introduced.

**Table 1:** Different types of textile electrode samples

No.	Textile electrode sample	Textile electrode composition and technology
1	HTL-1	Silver fiber shielding unidirectional elastic fabric (two sides)
2	HTL-2	Silver fiber shielding unidirectional elastic fabric (four sides)
3	LMF-2	Silver fiber shielding canvas fabric
4	ZZB-1 + 1	Silver fiber shielding knitted fabric
5	BTL-1 + 1	Silver fiber bidirectional elastic fabric (two sides)
6	KZZB	Antioxidant silver fiber knitted fabric
7	LXG-1	Silver fiber ripstop fabric
8	BTL-1	Silver fiber bidirectional elastic fabric (four sides)
9	ZZB-1	Silver fiber shielding knitted fabric
10	MZG-1	Silver fiber shielding ripstop fabric

As shown in Fig. 1, the textile electrode sample was formed by first sticking an insulating pad to the bottom of the conductive fabric sample and then fixing them with a metallic snap fastener.

As shown in Table 1, these textile electrode samples were woven from silver fibers with different weaving process.

Based on the textile electrode samples, the accuracy and range of the above feature parameters for the PEEP and AEEP were measured, including SEEC [including the skin–electrode static impedance (SESI), skin–electrode alternating current impedance (SEAI), and skin–electrode polarization voltage (SEPV)], SECP, SERM, and CLAS.

### Feature parameters based on electrochemical skin models

Based on the above electrochemical evaluation method, an evaluation platform based on an active simulated skin model and a passive simulated skin model was constructed, in which SEEC [including the skin–electrode static impedance (SESI), skin–electrode alternating current impedance (SEAI), and skin–electrode polarization voltage (SEPV)], SECP, SERM, and CLAS could be quantitatively measured for textile electrodes.

- Feature parameters extracted based on PEEP

Based on the PEEP, nine feature parameters were obtained, as shown in Table 2, and the parameter names, parameter codes, feature classifications, definitions, and measurement components are described in detail.

**Table 2:** Feature parameters extracted for the PEEP

No.	Parameter name	Parameter code	Feature classification	Parameter definition	Measuring device for the PEEP
1	Upper skin–electrode contact pressure	USECP	SECP	The contact pressure between electrode 1 and the upper Millipore film	Pressure device
2	Lower skin–electrode contact pressure	LSECP	SECP	The contact pressure between electrode 2 and the lower Millipore film	Pressure device
3	Upper skin–electrode relative moving speed	USERS	SERM	The skin–electrode relative moving speed between electrode 1 and the upper Millipore film	Pressure device
4	Lower skin–electrode relative moving speed	LSERS	SERM	The skin–electrode relative moving speed between electrode 2 and the lower Millipore film	Pressure device
5	Upper skin–electrode relative path of motion	USERPM	SERM	The skin–electrode relative path of motion between electrode 1 and the upper Millipore film	Pressure device
6	Lower skin–electrode relative path of motion	LSERPM	SERM	The skin–electrode relative movement between electrode 2 and the lower Millipore film	Pressure device
7	Static impedance of electrode pairs	SIEP	SEEC	The sum of the skin–electrode static impedance of electrode 1 and electrode 2 at the upper and lower Millipore films	Measuring device
8	Alternating current impedance of electrode pairs	ACIEP	SEEC	The sum of skin–electrode alternating current impedance of electrode 1 and electrode 2 at the upper and lower Millipore films	Measuring device
9	Polarization voltage of electrode pairs	PVEP	SEEC	The sum of skin–electrode polarization voltage of electrode 1 and electrode 2 at the upper and lower Millipore films	Measuring device

b. Feature parameters extracted based on AEEP

Based on the AEEP platform, 11 feature parameters were obtained, as shown in Table 3, and the parameter names, parameter codes, feature classifications, definitions, and measurement components are described in detail.

**Table 3:** Feature parameters extracted for the AEEP

No.	Parameter name	Parameter code	Feature classification	Parameter definition	Measuring device for the AEEP
1	Left skin–electrode contact pressure	LSECP	SECP	The contact pressure between electrode 1 and the left Millipore film	Pressure device
2	Right skin–electrode contact pressure	RSECP	SECP	The contact pressure between electrode 2 and the right Millipore film	Pressure device
3	Left skin–electrode relative moving speed	LSERS	SERM	The skin–electrode relative moving speed between electrode 1 and the left Millipore film	Pressure device
4	Right skin–electrode relative moving speed	RSERS	SERM	The skin–electrode relative moving speed between electrode 2 and the right Millipore film	Pressure device
5	Upper skin–electrode relative path of motion	USERPM	SERM	The skin–electrode relative path of motion between electrode 1 and the left Millipore film	Pressure device
6	Lower skin–electrode relative path of motion	LSERPM	SERM	The skin–electrode relative path of motion between electrode 2 and the right Millipore film	Pressure device
7	Static impedance of electrode pairs	SIEP	SEEC	The sum of the skin–electrode static impedance of electrode 1 and electrode 2 at the left and right Millipore films	Measuring device
8	Alternating current impedance of electrode pairs	ACIEP	SEEC	The sum of the skin–electrode Alternating current impedance of electrode 1 and electrode 2 at the left and right Millipore films	Measuring device
9	Polarization voltage of electrode pairs	PVEP	SEEC	The sum of the skin–electrode polarization voltage of electrode 1 and electrode 2 at the left and right Millipore films	Measuring device
10	Signals from the signal generator	SFSG	CLAS	Signals extracted by reference electrode 1 and reference electrode 2 from the signal generator	Measuring device
11	Signals extracted by measured electrodes	SFME	CLAS	Signals extracted by measured electrode 1 and measured electrode 2	Measuring device

### Skin–electrode electrochemical characteristic (SEEC)

The static impedance, alternating current impedance, and polarization voltage of textile electrodes were measured in the electrochemical evaluation platform.

In this experiment, SEEC parameters including static impedance, alternating current impedance, and polarization voltage were tested on the above 10 types of electrode samples.

**Fig. 2:**
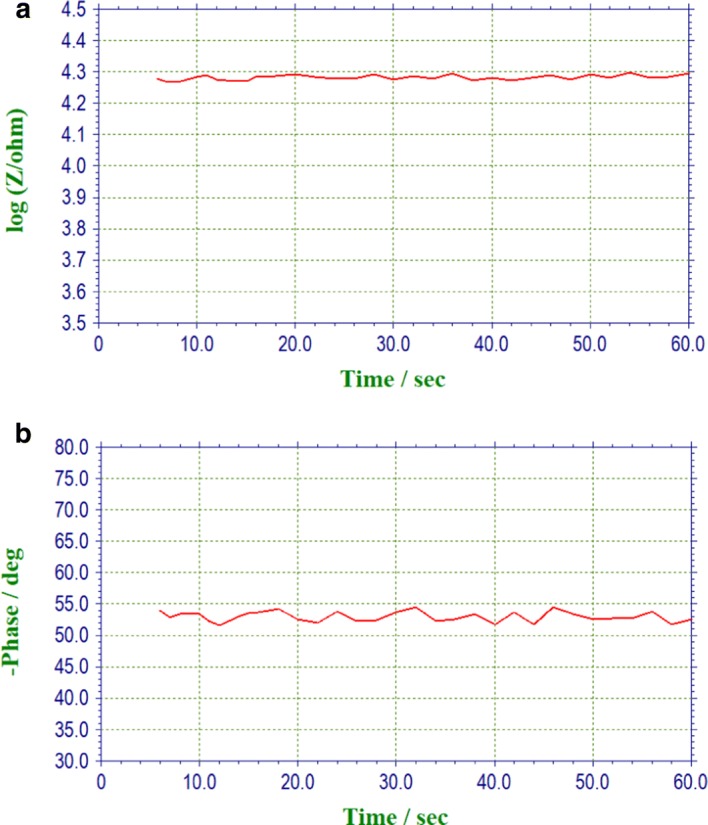
Static impedance curves for the HTL-1 textile electrode. **a** Impedance vs time curve. **b** Phase vs time curve

**Fig. 3:**
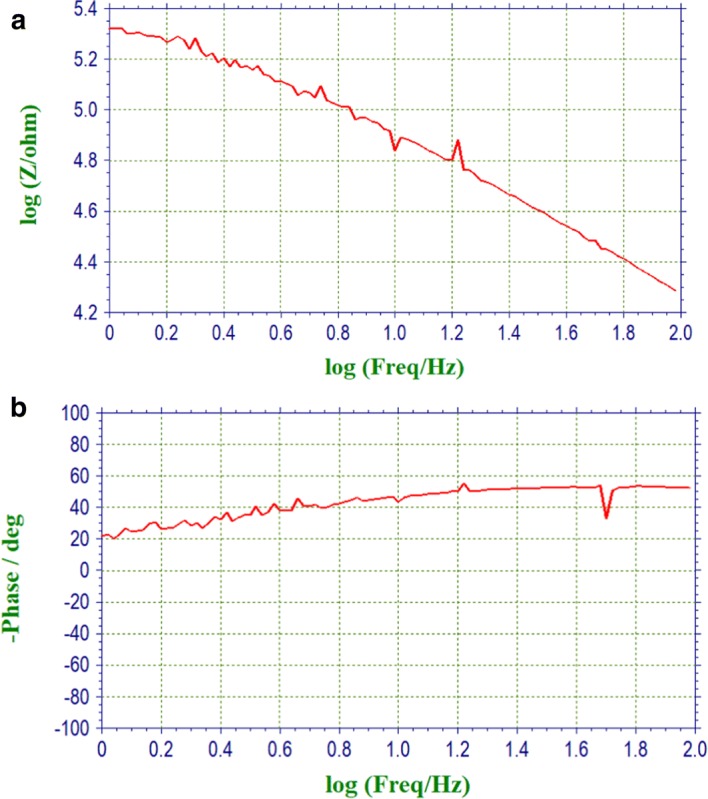
Alternating current impedance curves for the HTL-1 textile electrode. **a** Impedance vs frequency curve. **b** Phase vs frequency curve

**Fig. 4:**
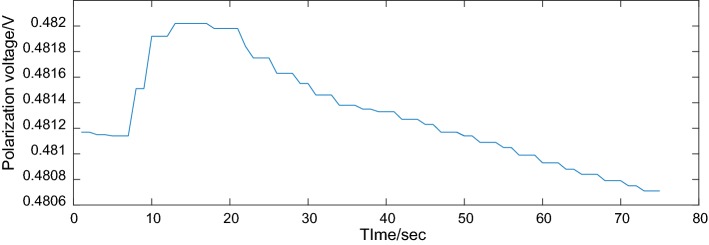
Polarization voltage curve for the HTL-1 textile electrode

Taking one type of textile electrode (HTL-1) as an example, the static impedance curve, alternating current impedance curve, and polarization voltage curve when USECP was 169.9 cN are shown separately in Figs. 2, 3 and 4.

As shown in Fig. 2, the data of impedance and phase were drawn, whose tolerance were ± 0.14 O and 1.85° separately. In this experiment, static impedance had different measured values for different contact pressures; however, for the setting contact pressure, the static impedance had a small tolerance after repeated measurement 10 times (depending on the performance of electrochemical workstations). For different types of textile electrodes, different static impedance values could be obtained for different setting skin–electrode contact pressure through this platform. These experimental data could be used to analyze the correlation among static impedance, skin–electrode contact pressure, and fabric process for textile electrodes.

As shown in Fig. 3, alternating current impedance curves for the HTL-1 textile electrode were drawn when USECP was 169.9 cN. As the frequency increases, the impedance value of textile electrode decreases. In this experiment, alternating current impedance had different measured values for different SECP; however, for the setting contact pressure, the alternating current impedance was very consistent after repeated measurement 10 times (depending on the performance of electrochemical workstations). For different types of textile electrodes, different alternating current impedance values could be obtained for different setting skin–electrode contact pressure through this platform. These experimental data could be used to analyze the correlation among alternating current impedance, skin–electrode contact pressure, and fabric process for textile electrodes.

On the other hand, different biomedical signal extraction for textile electrodes application has a great relationship with the alternating current impedance with different frequency bands. For example, if one type of textile electrode sample was used to collect electrocardiogram (ECG) signals, the alternating current impedance in the frequency domain (0.1–200 Hz) should be analyzed.

As shown in Fig. 4, the data of polarization voltage parameter obtained on the PEEP were accordant, whose tolerance was ± 0.65 mV. In this experiment, polarization voltage had different measured values for different contact pressures; however, for the setting contact pressure, the polarization voltage had a small tolerance after repeated measurement 10 times (depending on the performance of electrochemical workstations). For different types of textile electrodes, different polarization voltage values could be obtained for different setting skin–electrode contact pressure through this platform. These experimental data could be used to analyze the correlation among polarization voltage, skin–electrode contact pressure, and fabric process for textile electrodes.

### Accuracy and range of SECP parameters

In this research, the weights of measured electrode 1 and measured electrode 2 were fixed on the PEEP and AEEP. A pressure sensor was used to measure SECP parameters based on the PEEP and AEEP, whose accuracy and range were measured by weights with different weights.

**Fig. 5:**
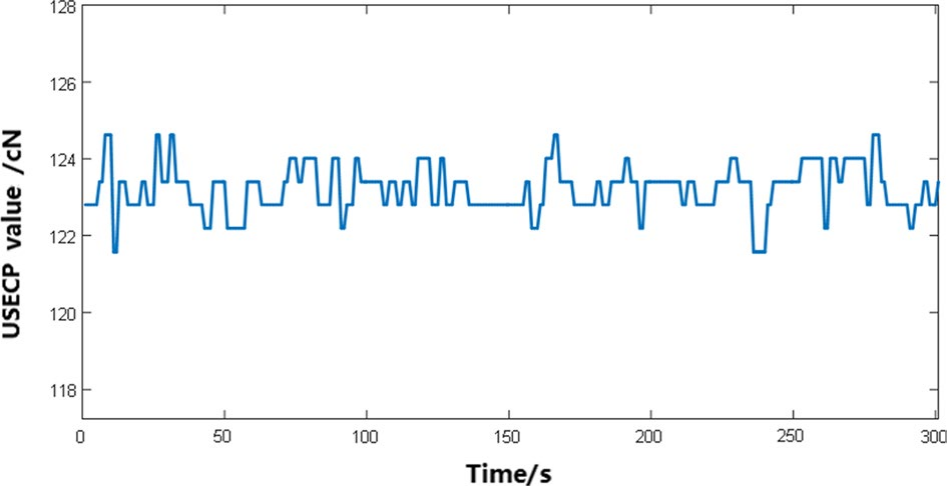
Upper skin–electrode contact pressure curve on the PEEP

The USECP parameter was measured at a setting value of 123 cN based on the PEEP for HTL-1 textile electrode sample, and the data curve is shown in Fig. 5.

As shown in Fig. 5, the data of USECP parameter obtained on the PEEP were accordant, whose tolerance was ± 1.8 cN. In this experiment, the contact pressure curves were tested on the above 10 types of electrode samples under the setting pressure of 123 cN, whose tolerance ranged from 1.1 to 5.3 cN.

Due to the weaving soft structure for textile electrodes, the contact pressure of the textile electrode to the surface of the human skin has a certain fluctuation range. Therefore, the fluctuation characteristics of this contact pressure should be taken into account when using textile electrodes.

### Accuracy and range of SERM parameters

The accuracy of SERM parameters based on the PEEP and AEEP was measured by comparing the difference between the setting and measured values. Experimental results showed that the accuracy of the relative movement was within 1 mm and the accuracy of the relative moving speed was within 1 mm/s.

The range of relative movement was 0–100 mm, and the range of relative moving speed was 0–40 mm/s.

### CLAS results

**Fig. 6:**
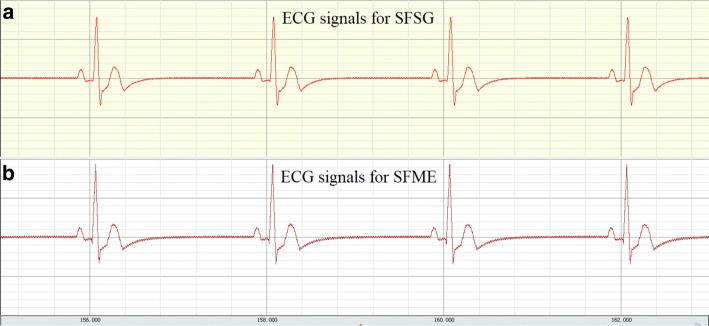
ECG signals from signal generator (SFSG) and measured electrodes (SFME). **a** ECG signals from signal generator. **b** ECG signals from measured electrodes

The SFSG and SFME on the AEEP based on an electrocardiogram (ECG) generator (equipment model: MPS450, FLUKE) are shown in Fig. 6. CLAS was defined as the difference in time domain and frequency domain between SFSG and SFME, and the performance of textile electrodes was evaluated.

As shown in Fig. 6, ECG signals from signal generator (SFSG) and measured electrodes (SFME) were similar in shape of the waveform, but there may be differences in information in time domain and frequency domain. For example, it was obvious that ECG signals for SFME had more noise interference than SFSG, which may provide an important basis for the application of textile electrodes.

Experimental results showed that the data obtained by the evaluation platform were effective and consistent after repeated measurement 10 times. And SEEC parameters were useful for the evaluation and application of textile electrodes.

### Evaluation for textile electrodes to skin

Correlation between SEEC and SECP parameters for 10 kinds of textile electrode samples based on PEEP was studied in this research. For alternating current impedance characteristics in SEEC, the following characteristics were extracted in the frequency range of 0–100 Hz, as shown in Table 4.

**Table 4:** Eleven alternating current impedance characteristics

No.	1	2	3	4	5	6	7	8	9	10	11
Frequency range (Hz)	0–1	1–2	2–3	3–4	4–5	5–10	10–20	20–30	30–40	40–50	50–100
Characteristics (Ω)	ACI-1	ACI-2	ACI-3	ACI-4	ACI-5	ACI-6	ACI-7	ACI-8	ACI-9	ACI-10	ACI-11

As shown in Table 4, ACI-1 represents the sum of the absolute ACI values in the frequency range 0–1 Hz.

Based on the 10 textile electrodes in Table 1, the correlation coefficients for 13 SEEC (i.e. static impedance and alternating current impedance) and 4 SECP (including USECP, LSECP, SECP_D, and SECP_S) characteristics were calculated and shown in Table 5.

**Table 5:** Correlation coefficients between electrochemical and SECP characteristics

No.	Correlation coefficients	USECP	LSECP	SECP_D	SECP_S
1	*Z*_se__static	− 0.5777 ± 0.2351	0.5705 ± 0.2285	0.5226 ± 0.3118	0.5680 ± 0.2108
2	*Φ*_se__static	0.5385 ± 0.4092	− 0.4763 ± 0.2595	− 0.6186 ± 0.1422	0.5187 ± 0.2108
3	ACI-1	− 0.8624 ± 0.1244^a^	0.5979 ± 0.2194	− 0.6051 ± 0.2570	− 0.5474 ± 0.2632
4	ACI-2	− 0.8322 ± 0.1800^a^	0.5873 ± 0.2173	0.5893 ± 0.2533	0.5407 ± 0.2761
5	ACI-3	− 0.8993 ± 0.1548^a^	0.5730 ± 0.2163	0.5872 ± 0.2523	0.5141 ± 0.2743
6	ACI-4	− 0.8887 ± 0.1455^a^	0.5701 ± 0.2165	0.5736 ± 0.2547	0.5120 ± 0.2793
7	ACI-5	− 0.8959 ± 0.1413^a^	0.6089 ± 0.2163	0.6124 ± 0.2558	0.5525 ± 0.2770
8	ACI-6	− 0.8901 ± 0.1303^a^	0.5711 ± 0.2155	0.5796 ± 0.2595	0.5207 ± 0.2832
9	ACI-7	− 0.7808 ± 0.1296	0.5063 ± 0.2143	0.5232 ± 0.2621	0.4898 ± 0.2869
10	ACI-8	− 0.7457 ± 0.1265	0.4618 ± 0.2148	0.5395 ± 0.2620	0.4656 ± 0.2894
11	ACI-9	− 0.7508 ± 0.1244	0.4298 ± 0.2152	0.4300 ± 0.2616	0.4724 ± 0.2908
12	ACI-10	− 0.7205 ± 0.1234	0.4696 ± 0.2156	0.4523 ± 0.2613	0.4973 ± 0.2917
13	ACI-11	− 0.7112 ± 0.1206	0.5052 ± 0.2164	0.4975 ± 0.2608	0.5083 ± 0.2935

^a^The significant correlation (*t* = 0.01); the definition of USECP and LSECP are shown in Table 2; the difference between USECP and LSECP is denoted by SECP_D; SECP_S denotes the sum of USECP and LSECP (SECP_S); *Z*_se__static is skin–electrode static impedance; *Φ*_se__static is skin–electrode static phase

Experimental results show that correlation coefficients between electrochemical characteristics and USECP are all above 0.5, which are all negatively correlated. Means and variances of correlation coefficients based on the 10 textile electrode samples in Table 1 are shown in Table 5.

As shown in Table 5, there was no significant correlation of *Z*_se__static and *Φ*_se__static with SECP characteristics.

**Fig. 7:**
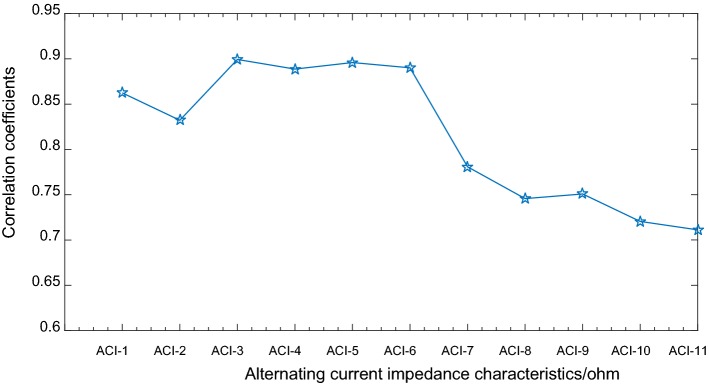
Correlation coefficient curve for ACI characteristics and USECP

Correlation coefficients between ACI-1, ACI-2, ACI-3, ACI-4, ACI-5, ACI-6 and USECP are higher (above 0.8) with significant correlation. That is, ACI spectra below 10 Hz had a significant negative correlation with the SECP values, reaching the highest value in the 2–3 Hz band (‘ACI_3’), and the correlation coefficients below 10 Hz were higher than those above 10 Hz. Moreover, the correlation coefficients above 10 Hz showed a downward trend, as shown in Fig. 7.

As shown in Table 5, the correlation coefficients for the ACI characteristics and LSECP, SECP_D, and SECP_S are smaller than those for USECP. In addition, the values at approximately 0.5 Hz do not have a significant correlation. However, one consistent trend was found for LSECP, SECP_D, and SECP_S that was the same as that observed for USECP; i.e., the correlation coefficients below 10 Hz were higher than those above 10 Hz.

These experimental results show that alternating current impedance below 10 Hz for textile electrodes has a great correlation with skin–electrode contact pressure. If textile electrodes are used to collect biomedical signals below 10 Hz (such as ECG, EEG) from human body, the consistency of contact pressure between electrode and the skin should be maintained. The skin–electrode contact pressure has a large correlation with the alternating current impedance below 10 Hz. If textile electrodes are used to collect an effective signal above 10 Hz (such as EMG), a good contact with the skin should be kept, and electrode–skin contact pressure and alternating current impedance above 10 Hz have a less correlation.

**Fig. 8:**
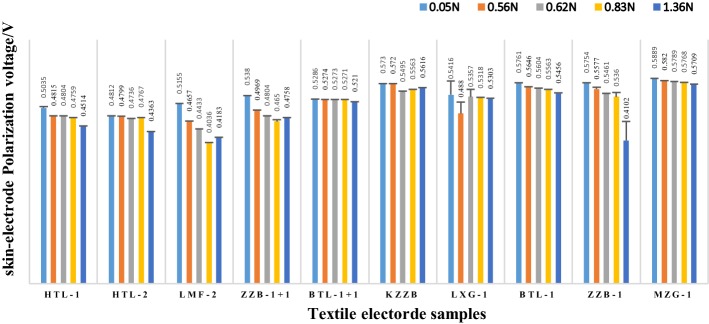
Skin–electrode polarization voltage vs pressure value for different textile electrode samples

On the other hand, the correlation between skin–electrode polarization voltage (SEPV, denoted by *U*_p-se_) and skin–electrode contact pressure (SECP) was analyzed based on 10 types of textile electrodes shown in Table 1. Five contact pressure sample points were selected, which were 0.05 N, 0.56 N, 0.62 N, 0.83 N, 1.36 N. Based on these five contact pressure values, skin–electrode polarization voltages were performed on 10 fabric electrode samples as shown in Fig. 8.

It can be seen in Fig. 8 that the polarization voltages of most textile electrode samples show a downward trend with the increase of contact pressure, but the magnitude of the decrease is not very large, which means that when using textile electrodes for bioelectrical signal extraction, a good contact should be kept as much as possible. A better contact between textile electrode and the human skin can make a smaller polarization voltage, but the textile electrode and the skin do not need to be too close, because the reduction in polarization voltage is not very obvious; on the contrary, it may cause human feel uncomfortable.

**Fig. 9:**
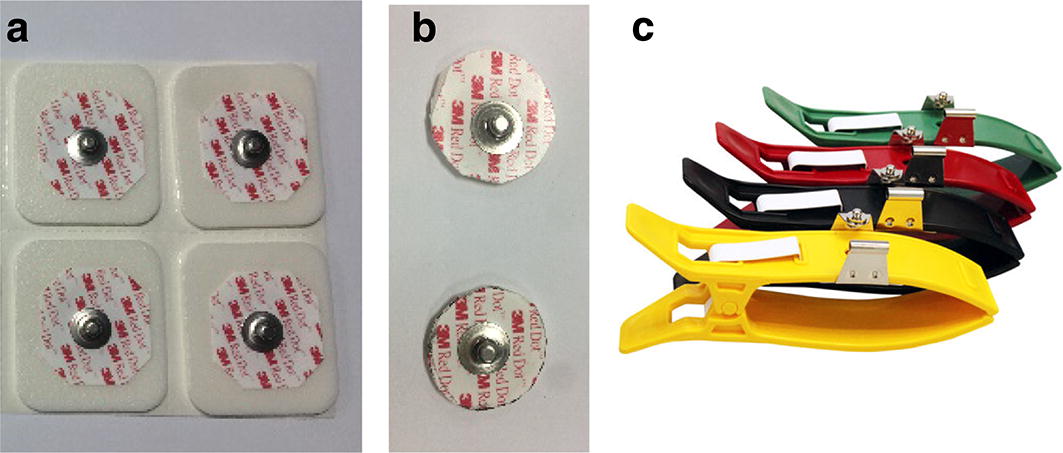
Traditional paste ECG electrodes in hospitals. **a** 3M ECG electrode, **b** 3M ECG electrode with 2 cm diameter, **c** stainless-steel electrode

To evaluate textile electrodes, some standard ECG electrodes in hospitals were selected in this research, which included 3M ECG electrode, 3M ECG electrode with a diameter 2 cm, and stainless-steel electrode shown in Fig. 9. Among them, the model of 3M ECG electrode was 3M 2560, and 3M ECG electrode sheared to a circle of 2 cm in diameter was analyzed and shown in Fig. 9b. In addition, a stainless-steel electrode (model: REZ028B) made in Heal Force Bio-meditech Holdings Limited was analyzed.

**Fig. 10:**
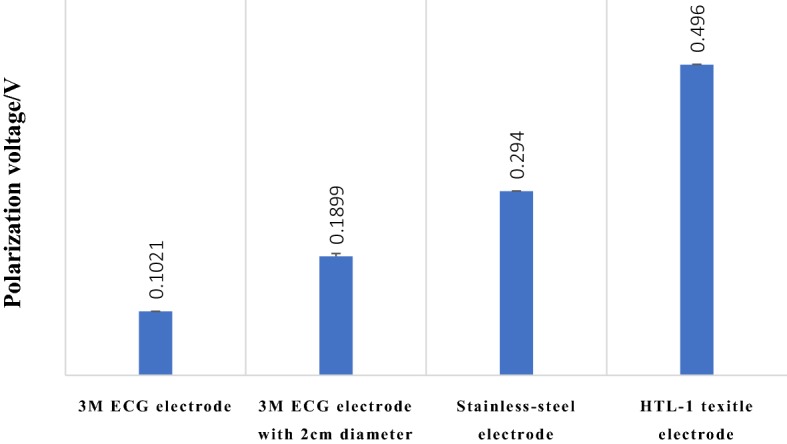
Polarization voltages for different electrodes

Means and variances of polarization voltages for traditional pasted ECG electrodes and HTL-1 textile electrode are shown in Fig. 10.

As shown in Fig. 10, polarization voltage for HTL-1 textile electrode was bigger than 3M ECG electrodes.

## Discussion

### The reasons for this research

The reasons why we studied the skin–electrode electrochemical interface for wearable textile electrodes are shown as follows:

Wearable dry biomedical electrodes, such as textile electrodes, noncontact electrodes, and microneedle electrodes, are one type of wearable sensor technologies and have been studied by an increasing number of researchers [18–26]. However, some problems in application of dry electrodes have occurred.

Dry electrodes were used to extract physiological signals from human body without conductive paste, which cause that electrodes did not touch the skin very well. Compared with wet electrodes, the skin–electrode interface characteristics for dry electrodes have bigger differences. First of all, the contact impedance and polarization voltage in skin–electrode interface for dry electrodes increase greatly, which bring extracted physiological signals with more noise, and weaken useful physiological signals at the same time [29]. Second, the relative displacement between the electrode and the skin for dry electrodes often occurs, which usually results in changes in extracted biomedical signals [17]. Third, a good contact between the dry electrode and the skin is good for a relatively stable physiological waveform. When the contact between the dry electrode and the skin becomes loose, the signal will be greatly weakened. How much pressure could meet the demand of signal extraction? On the contrary, too much contact pressure could make people feel uncomfortable. Therefore, the differences between wet electrodes and dry electrodes for skin–electrode interface characteristics are the point in this research, which will be of great help to the application and development of electrodes.

### Simulated skin model

Is it necessary to set up a simulated skin model? The answer is yes.

For dry electrode developers, it is a problem to evaluate dry electrodes. The method they used was usually the performance measurement including the impedance and polarization voltage for electrode pairs, and the biomedical signals were extracted by the impedance matching circuit and the conditioning amplifier circuit. However, when the dry electrode was used to collect the physiological signals from the body surface, it was found that the signal was weak or even could not be extracted.

In fact, the reason for the above phenomenon is that electrochemical characteristics for dry electrode and skin interface were unknown. Different human bodies have different skin characteristics, so that one type of dry electrodes can pick up a complete ECG waveform from some people while can do nothing about others. Based on the above analysis, it is necessary to establish a standard skin model to evaluate the different characteristics of different dry electrodes, which is of great help to the development of dry electrodes.

**Fig. 11:**
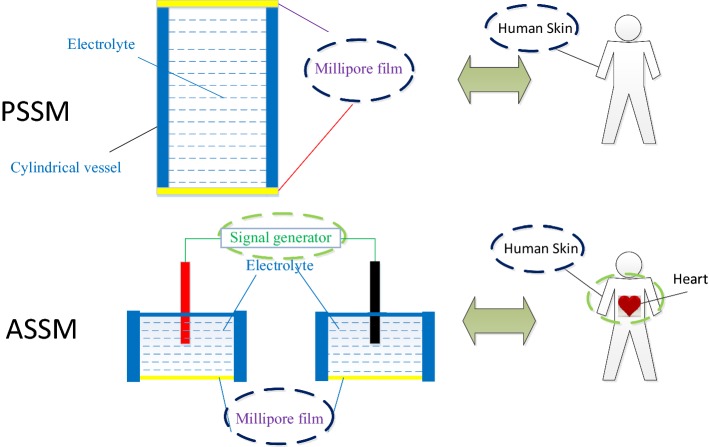
Correlation between electrochemical skin modeling and human skin. **a** PSSM, **b** ASSM

Two electrochemical skin models including an active simulated skin model and a passive simulated skin model were established using Millipore films in this research, and Millipore films were used to simulate human skin as shown in Fig. 11.

The passive simulated skin models (PSSM) were mainly used to analyze the electrochemical characters in the skin–electrode interface, while active simulated skin models (ASSM) could simulate the beating of the human heart and physiological signals could be extracted from the “skin” surface. And the signal generator on ASSM was used to simulate human heart, and the signal was transmitted from the generator to the surface of the film, which was the same as the ECG signal from the heart to the surface of the human skin.

Millipore films have hydrophilic and different pore size. Researchers could choose different pore sizes according to different needs, and the electrolytes in cylindrical vessel could be water, saline or ECG conductive paste according to different needs.

### Evaluation methods

Chinese YY/T pharmaceutical industry standard YY/T 0196-2005 (disposable ECG electrode) says the mark, the safety, and the performance requirements of the disposable ECG electrode used in ECG diagnosis and monitoring [17]. It says that any disposable ECG electrode system consisting of sensor elements and electrolytes is included in this standard. While, active electrodes, needle electrodes, reusable electrodes, and electrodes for energy transferring are not included in the standard range. DC offset impedance indicator is defined as the charge storage capacity (capacitance) of preventing current flowing through the electrode interface (resistance) in response to sinusoidal current. For alternating current impedance indicator, the impedance average should not exceed 2 kΩ among 12 pairs of adhesive electrodes at 10 Hz and no more than 100 uA (peak–peak) current. The impedance for each pair of adhesive electrodes should not exceed 3 kΩ.

According to the standard, the performance of disposable electrodes is often tested through pairs of electrodes [17]. However, this method is no longer applicable for new textile electrodes. Textile electrodes are dry electrodes that do not require pretreatment of the skin or the use of a conductive paste when extracting surface bioelectrical signals from the body. This trait determines the difference in impedance and offset voltage between wet and dry electrodes. In addition, the characteristics of the electrode–skin interface for textile electrodes model the exchange of information in extracting bioelectric signals from the surface of the human body.

Based on above reasons for this research, three evaluation methods were proposed as shown in Fig. 17, and the researchers could choose the suitable evaluation method according to their different application requirements. The first method was used to evaluate the correlation between SEEC and skin–electrode contact pressure (SECP), with whom the suitable SECP values were selected in the wearable application of dry electrodes. The second method can evaluate the correlation between SEEC and skin–electrode relative movement (SERM), with whom researchers could know how to control the displacement of dry electrodes to skin, and know the impact of displacement on signal acquisition by dry electrodes. The third method was applied to evaluating the difference between the signal source and the output signal, with which researchers could analyze the difference of physiological signals extracted by dry electrodes in different frequency bands.

### Evaluation platform

Based on the methods in this research, different instruments can be selected to set up the evaluation platform. As long as the equipment precision is well controlled, the electrochemical characters of the textile electrode could be acquired. And the simulated skin model in the evaluation platform could be made into an arc shape, which could better simulate the characteristics of skin surface.

In addition, the pressure-measuring equipment in the evaluation platform should be built with good accuracy, because the pressure performance for different dry electrodes on the skin surface will be different due to the different manufacturing process. And electrochemical measuring instruments in the evaluation platform could be selected from other models from different companies, as long as they can accurately extract the impedance and polarization voltage. The signal generator on AEEP could be not only a 1-mV standard signal generator but also a physiological signal generator, such as EEG signal generator and ECG signal generator.

### Evaluation for textile electrode samples

In this research, 10 textile electrode samples were produced with different plating processes and different textile processes. Based on these samples, ten USECP values between 0 and 3 N were selected for each textile electrode to control the relative position between electrode 1 and the upper Millipore film. Fourteen electrochemical characteristics were collected at each USECP value.

The experimental results showed significant correlations between six electrochemical characteristics and USECP for the textile electrodes. That is, ACI spectra below 10 Hz had a significant negative correlation with USECP values, reaching the highest value in the 2–3 Hz band, and correlation coefficients below 10 Hz were higher than those above 10 Hz. Moreover, correlation coefficients above 10 Hz showed a downward trend.

As shown in Table 5, compared with LSECP, SECP_D, and SECP_S, the correlation coefficient between ACI characteristics and USECP is the highest; that is, the correlation between the ACI characteristics and the contact pressure in the upper Millipore film is higher than that for other contact pressure values. This difference is because the relative position between electrode 1 and the upper Millipore film changes with USECP, while the relative position of electrode 2 and the lower Millipore film does not change. The correlation coefficients between ACI and SECP on the lower surface are near 0.5, and the correlation is not significant. The results indicate a strong correlation between ACI and SECP in textile electrodes.

Based on characteristics extracted for ACI absolute values, the correlation between the ACI phase characteristics and SECP was also analyzed in this research. Experimental results showed that the correlation between the ACI phase characteristics and SECP was not significant, and the correlation coefficients were near 0.5 and lower.

On the other hand, the correlation between polarization voltage and skin–electrode contact pressure was analyzed based on 10 types of textile electrodes. Experimental results showed the polarization voltages of most textile electrode samples show a downward trend with the increase of contact pressure, but the magnitude of the decrease is not very large, which means that when using textile electrodes for bioelectrical signal extraction, a good contact should be kept as much as possible. A better contact between textile electrode and the human skin can make a smaller polarization voltage, but the textile electrode and the skin do not need to be too close.

## Conclusions

Wearable textile electrodes have been accepted and applied by increasing numbers of people [27–30], and electrochemical characteristics of the skin–electrode interface were proposed to evaluate textile electrodes to skin, which was the innovation of this research. Two SEEI models for textile electrodes were proposed in this research, including a single transient model and double transient model, and three SEECs, including SESI, SEAI, and SEPV, were proposed and applied to the evaluation of textile electrodes, and then three evaluation methods for textile electrodes were proposed, including the correlation between SEEC and SECP, SERM, and CLAS.

An evaluation platform based on an ASSM and a PSSM was constructed, in which some electrochemical characteristics, including SEEC, SECP, SERM, and CLAS, for textile electrodes were quantitatively measured, and these characteristics could be used to evaluate other bioelectrical electrodes, such as noncontact electrodes and microneedle electrodes. Based on the PEEP, 11 feature parameters were obtained, and the parameter names, parameter codes, feature classifications, definitions, and measurement components are described in Table 1. The PEEP could provide a vivid means of analyzing the correlation among the concentration of electrolytes in the cylinder, cylinder length, skin-simulating film size, and so on. Based on the AEEP 13 feature parameters were obtained, and the parameter names, parameter codes, feature classification, definitions, and measurement components are described in Table 3. The AEEP in this research could be used to evaluate electrode performance quantitatively, which was composed of three parts: an active simulated skin module, a pressure-controlling device, and a measuring device. In AEEP, Millipore films were used to simulate human skin, a signal generator was used to simulate the heart, an electrolyte was used to simulate the tissue, and an electrode–skin interface was established. The signal from the signal generator passed through the electrolyte, Millipore films, and electrodes, which was the same as biomedical signals generated from the heart pass through the tissue, the skin, and an electrode.

Finally, the accuracies of the above feature parameters, including SECP, SERM, SEEC, and CLAS, were measured in the PEEP and AEEP. The experimental results showed that the data obtained by the testing platform were effective, and this platform could provide strong support for the performance evaluation of textile electrodes to skin. These methods in this research were also effective in evaluating other dry electrodes, such as 3M electrode, stainless-steel electrode, dry electrode, and microneedle electrode.

## Methods

### Skin–electrode electrochemical interface (SEEI)

**Fig. 12:**
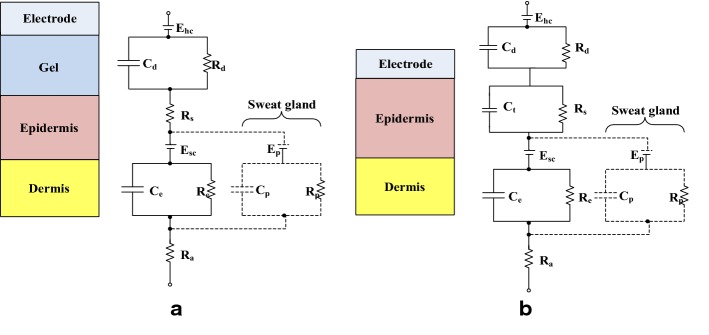
Equivalent circuit of skin–electrode electrochemical interface for (**a**) traditional wet electrodes and (**b**) textile electrodes. *E*_hc_ is the electrode half-cell potential between the electrode and the gel; *C*_d_ is the equivalent capacitance due to electrical double-layer information; *R*_d_ is charge transfer resistance; *R*_s_ is a series resistance of the gel medium; *E*_sc_ and *E*_p_ are the inductive potential of the skin–electrode interface and a sweat gland; *C*_e_ and *R*_e_ are the equivalent capacitance and resistance of the epidermis; *E*_p_, *C*_p_, and *R*_p_ stand for the effect of sweat glands as a parallel conduction path through the epidermis; *R*_a_ is a pure resistance of the dermis

Human skin has a complex multilayer structure, which consists of three main layers: epidermis, dermis and hypodermis, and the epidermis plays an important role in the skin–electrode interface. Different skins from different people have different electrochemical characteristics including skin–electrode impedance and its frequency-dependent characteristics. An electrode–electrolyte interface can be modeled by a parallel RC network, and an equivalent circuit of skin–electrode electrochemical interface (SEEI) for traditional wet electrodes and textile electrodes is shown in Fig. 12 [15, 16]. The upper layer of the epidermis behaves as a semipermeable membrane that causes the difference in ion concentration and a potential difference, which is shown with *E*_hc_.

For textile electrodes, there is a strong capacitive behavior compared to conventional electrodes because of the absence of electrolytes. As shown in Fig. 12b, the effect with capacitance *C*_t_ plays an important role in the process of sensing biomedical signals for textile electrodes, and Ct is in inverse proportion with sweat and the moisture over the skin. Parallel RC blocks on the equivalent circuit imply skin–electrode contact impedance (SECI) decreases with increased frequency, and decreasing the SECI improves the signal quality.

### Simulated skin models (SSM)

Two simulated skin models were proposed in this research, including a passive simulated skin model (PSSM) and an active simulated skin model (ASSM).

- Passive simulated skin model (PSSM)

**Fig. 13:**
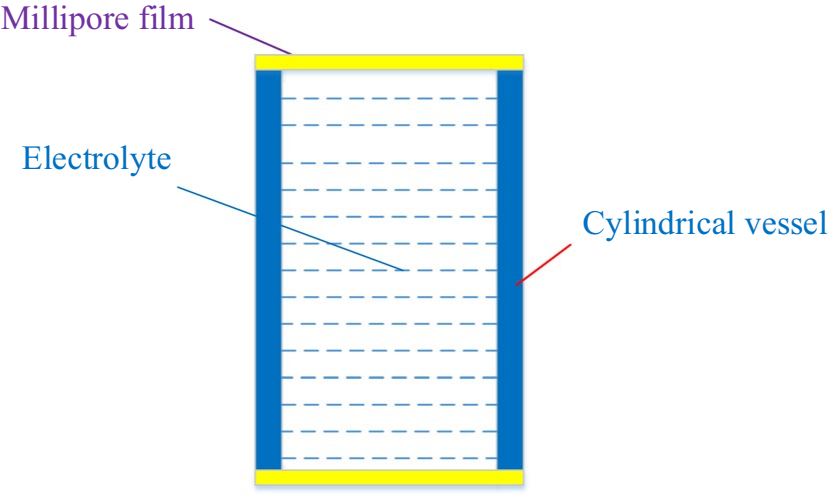
The design principle of the PSSM

The design model of the PSSM is shown in Fig. 13; this model is composed of one cylindrical vessel full of electrolytes and two hydrophilic films made by Millipore Company (named as Millipore film). A colloidal electrolyte or a liquid electrolyte is used to simulate human skin tissue fluid, the electrolyte is then placed into a cylindrical vessel, and the openings on the top and bottom ends of the cylindrical vessel are sealed with hydrophilic films.

The Millipore film is used as human simulated skin, and the electrolyte is used as the simulated tissue fluid. Different films and electrolyte have different skin–electrode electrochemical models. The electrolyte is usually replaced by medical conductive paste, NaCl electrolyte, pure water, and so on.

b. Active simulated skin model (ASSM)

**Fig. 14:**
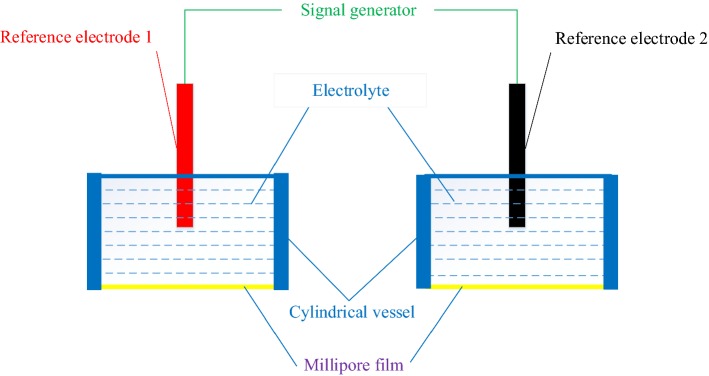
The design principle of the ASSM

Based two PSSMs and a signal generator, an active simulated skin model (ASSM) was constructed as shown in Fig. 14.

The active simulated skin module consists of two cylindrical vessels full of electrolytes, two Millipore films, two reference electrodes (reference electrode 1 and reference electrode 2), and one signal generator. As the same principle of PSSM, colloidal electrolytes or liquid electrolytes are used to simulate human skin tissue, and the electrolyte is placed in a cylindrical vessel, and the openings on bottom ends of two cylindrical vessels are sealed with Millipore films separately. In addition, two reference electrodes are inserted on top ends of two cylindrical vessels separately, and the two ends of the signal generator are connected with electrode 1 and electrode 2, respectively. And a signal from the signal generator is introduced into the electrolyte.

### Electrochemical evaluation platform (EEP)

Two electrochemical evaluation platforms based on PSSM and ASSM were proposed in this research, including a passive electrochemical evaluation platform (PEEP) and an active electrochemical evaluation platform model (AEEP).

- Passive electrochemical evaluation platform (PEEP)

**Fig. 15:**
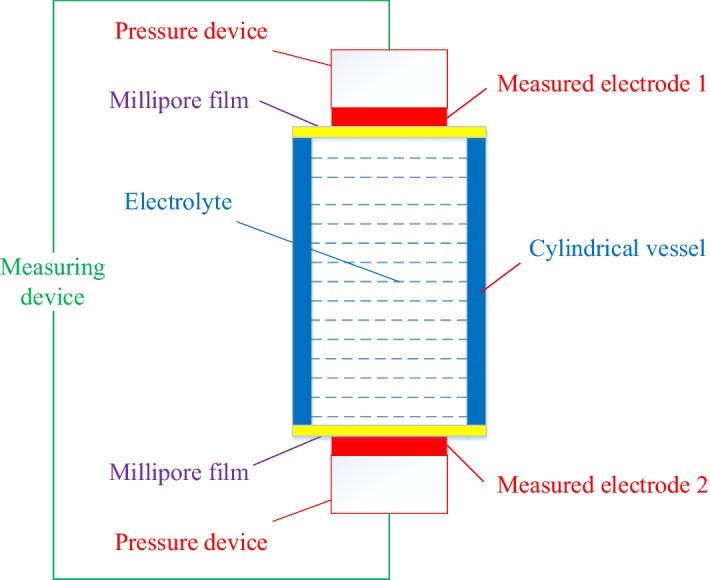
The design principle of the PEEP

Based on PSSM, a passive electrochemical evaluation platform (PEEP) is constructed as shown in Fig. 15, which consists a PSSM, two pressure devices, and sone measuring devices. Two measured electrodes are placed on the top and the bottom Millipore films for PSSM separately with two pressure devices, which can control the contact between the electrode and the film at a fixed pressure value.

Some relevant parameters for a pair of measured electrodes are obtained by measuring devices, including impedance spectra, static voltage, and dynamic open circuit voltage. Furthermore, some parameters can be adjusted with the platform, and these parameters include the concentration of electrolyte, skin-membrane pore size, skin-membrane electrode pressure, cylinder length, relative moving speed of the electrodes to the skin membrane, and relative path of motion between the electrodes and skin membrane.

b. Active electrochemical evaluation platform (AEEP)

**Fig. 16:**
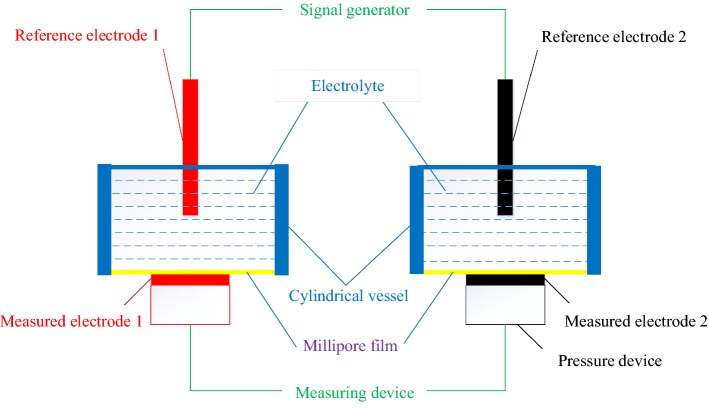
The design principle of the ASSM

Based on ASSM, an active electrochemical evaluation platform (AEEP) is constructed as shown in Fig. 16, which consists an ASSM, two pressure devices, and sone measuring devices. Two measured electrodes are placed on two bottom Millipore films for ASSM separately with two pressure devices, which can control the contact between the electrode and the film at a fixed pressure value.

The signal generator for ASSM could be used to simulate human hearts or heads such as Sine signals between 0.1 and 200 Hz for electrocardiogram (ECG), Sine signals between 0.05 and 30 Hz for electroencephalograph (EEG), or electrocardiogram (ECG) generator (equipment model: MPS450, FLUKE). Moreover, measured electrodes are placed on the skin surface with the pressure device, and the relevant parameters are obtained with measuring devices, including impedance spectra, static voltage, dynamic open circuit voltage, and signal-to-noise ratio. Furthermore, some parameters can be adjusted in the platform, and these parameters include the concentration of electrolyte, skin-membrane pore size, skin-membrane electrode pressure, cylinder length, speed of the electrode relative to the skin membrane, and relative path of motion between the electrode and skin membrane.

### Electrochemical evaluation methods

Chinese YY/T pharmaceutical industry standard YY/T 0196-2005 (disposable ECG electrodes) defines the labeling, safety, and performance requirements of disposable ECG electrodes used in ECG diagnosis and monitoring [17]. All disposable ECG electrode systems consisting of sensor elements and electrolytes are included in this standard. However, active electrodes, needle electrodes, reusable electrodes, and electrodes for energy transfer are not included in the scope of the standard.

The performance of disposable electrodes is often tested through pairs of electrodes. However, this method is no longer applicable for new textile electrodes. Textile electrodes are dry electrodes that do not require pretreatment of the skin or the use of a conductive paste when extracting bioelectrical signals from the surface of the body. This trait determines the difference in impedance and offset voltage between wet and dry electrodes. Moreover, the characteristics of electrode–skin interfaces for textile electrodes model the exchange of information in extracting bioelectric signals from the surface of the human body. Based on the SEEI models and YY/T 0196-2005, three skin–electrode electrochemical characteristics (SEECs), including skin–electrode static impedance (SESI, denoted by *Z*_se__static), skin–electrode alternating current impedance (SEAI, denoted by *Z*_se_), and skin–electrode polarization voltage (SEPV, denoted by *U*_p-se_), were applied to evaluate textile electrodes.

Some problems occur in the extraction of biomedical signals from the human skin surface for wearable health monitoring. First, when textile electrodes are used, the contact pressure between the electrode and the skin is large, and the signal is good. However, if the contact pressure is too large, the electrode may feel uncomfortable. Therefore, the amount of pressure applied for textile electrodes should be studied to determine the range that not only feels comfortable but also produces a good signal. Therefore, the correlation between SEEC and skin–electrode contact pressure (SECP, denoted by *F*_se_) should be analyzed, which is the first evaluation method (Method 1).

When textile electrodes are used, the movement of the human body inevitably causes relative movement between the textile electrode and the skin. The relative movement between the electrode and the skin often adds noise to the extraction of physiological signals. Therefore, the amount of movement that does not affect signal extraction needs further research. Thus, the correlation between SEEC and skin–electrode relative movement (SERM) should be analyzed, which is the second evaluation method (Method 2).

When textile electrodes are used to extract physiological signals from the human body, the differences between electrochemical characteristics in the input signal and in the output signal should be studied, which will provide important technical support for the application of textile electrodes. In addition, if one specific signal source (such as a signal generator or ECG simulator) is used, the output signals are extracted by textile electrodes through the simulated skin tissue and skin–electrode interface. Quantitatively comparing the difference between the signal source and the output signal [identified as the conduction loss of active signal (CLAS)] is the third evaluation method (Method 3).

**Fig. 17:**
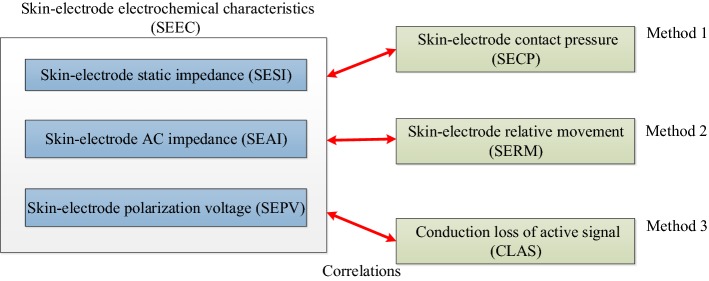
Evaluation methods for textile electrodes

The above three evaluation methods are represented in Fig. 17.

### Evaluation platform setup

**Fig. 18:**
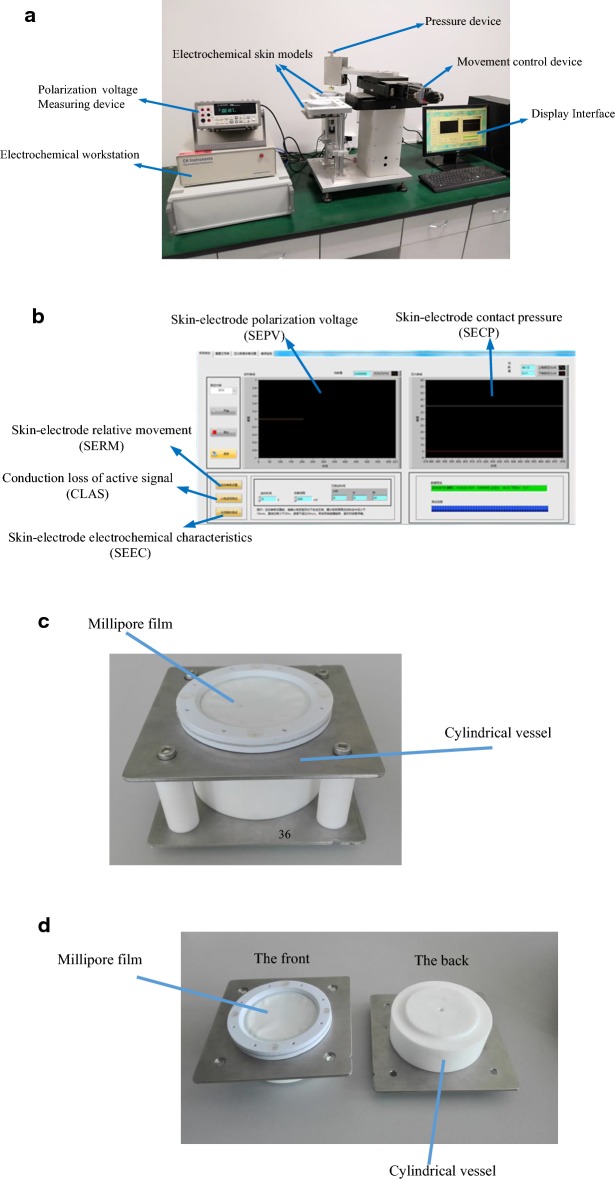
Images of evaluation platform in this research. **a** Whole platform, **b** display interface, **c** cylindrical vessel for PSSM, **d** cylindrical vessel for ASSM

The evaluation platform for textile electrodes was set up as shown in Fig. 18a, which consisted of movement control device, pressure device, Electrochemical skin models, polarization voltage measuring device, electrochemical workstation, and display interface. And the display interface for this platform is shown in Fig. 18b.

As shown in Fig. 18a, movement control device was used to control skin–electrode relative movement, and pressure device was used to extract skin–electrode contact pressure parameter. The polarization voltage-measuring device was used to extract skin–electrode polarization voltage parameter, and electrochemical workstation was used to extract skin–electrode static impedance and alternating current (AC) impedance. As shown in Fig. 18b, the display and control software for the PEEP and AEEP was designed, and feature parameters could be displayed and saved in display interface, which included skin–electrode polarization voltage, skin–electrode contact pressure, skin–electrode relative movement, the conduction loss of active signal, and skin–electrode electrochemical characteristics.

The film was selected from Millipore’s hydrophilic film with an effective pore size of 0.1 μm, a thickness of 0.135 mm and a diameter of 143 mm. Two pieces of ABS board (thickness: 5 mm, external diameter: 100 mm, internal diameter: 73 mm) were used to simulate the skin clamping, and in the following piece of ABS board there was groove with a diameter of 78 mm for the placement of seals, and two ABS boards were fixed with nylon screws.

The pressure devices on the platform could adjust pressure values between the electrode and the stimulated skin, and the direction was controlled by the rotary knob (clockwise for rising, counterclockwise for decreasing). The pressure value between the electrode and the skin could be measured using a pressure sensor (model: CD17-600g, the measurement range: 0–600 cN, accuracy: 0.1 cN, China).

The precision of the movement control device was within 1 mm, and the range of relative displacement was 0–100 mm. An electrochemical workstation (equipment model: CHI660 by Beijing Chinese science days Technology Co., Ltd.) as a measuring device was used to measure SEEC feature parameters of textile electrodes. And the main conditions were as follows:

- Electrochemical methods: cyclic voltammetry (CV), linear sweep voltammetry (LSV), sweep-step functions (SSFs), alternating current impedance (IMP), impedance-time (IMPT), impedance-potential (IMPE), chronopotentiometry (CP), etc.
- Sampling rate: 500 kHz
- Potential resolution: 0.1 mV
- Reference electrode input impedance: 10^12^ Ω
- Parameters: static impedance, alternating current impedance, polarization voltage, etc.

## Data Availability

The datasets used and/or analyzed during the current study are available from the corresponding author on reasonable request.

## Abbreviations

ECG: Electrocardiogram
EEG: Electroencephalogram
EOG: Electro-oculogram
EMG: Electromyogram
SEEI: Skin–electrode electrochemical interface
SEEC: Skin–electrode electrochemical characteristics
SESI: Skin–electrode static impedance
SEAI: Skin–electrode alternating current impedance
SEPV: Skin–electrode polarization voltage
SECP: Skin–electrode contact pressure
SERM: Skin–electrode relative movement
CLAS: Conduction loss of active signals
ASSM: Active simulated skin model
PSSM: Passive simulated skin model
EEP: Electrochemical evaluation platform
PEEP: Passive electrochemical evaluation platform
AEEP: Active electrochemical evaluation platform
